# Facilitating interoperability in manufacturing systems using digital twins: a multi-asset AAS system

**DOI:** 10.1007/s00170-025-17178-z

**Published:** 2025-12-21

**Authors:** Basem Elshafei, Jack C. Chaplin, David Sanderson, Svetan Ratchev

**Affiliations:** Institute for Advanced Manufacturing, University of Nottingham, University Park, Nottingham, NG7 2RD UK

**Keywords:** Industry 4.0, Asset Administration Shell (AAS), Digital twins, Data exchange

## Abstract

The Asset Administration Shell (AAS) has emerged as a key enabler for structured data representation and bidirectional data exchange between manufacturing assets and their corresponding digital representations, thereby implementing Digital Twins (DTs) within the framework of Industry 4.0 and the Reference Architecture Model Industrie 4.0. Despite its potential, bidirectional data exchange remains underexplored. Most practical applications are limited to unidirectional data collection, known as a ’Digital Shadow’ (DS) of the asset. Additionally, existing implementations typically focus on a single asset, which hinders interoperability, a critical requirement for smart manufacturing ecosystems. This study bridges these gaps by developing an AAS system that enables real-time monitoring and control across multiple assets through seamless communication, coordination, and orchestration. This research demonstrates and validates the capabilities of the AAS using three types of assets: a Mobile Industrial Robot (MiR-250), two KUKA KR4 R600 robots, and a KUKA KR1000 Titan robot. The implemented AAS framework retrieves data from these assets to monitor critical parameters while issuing control commands based on specific data conditions. The approach enables data exchange and coordinated interaction among the digital representations of assets (i.e., the AASs), effectively facilitating communication among the physical assets in interconnected, intelligent manufacturing systems. This research advances the role of AAS in achieving real-time communication and coordination within and between assets, addressing the persistent challenge of interoperability in multi-asset smart manufacturing environments. Our approach overcomes the limitations of unidirectional single-asset AAS implementations, paving the way for scalable, standardised, and autonomous Industry 4.0 ecosystems.

## Introduction

The manufacturing sector is undergoing a significant transformation driven by Industry 4.0 principles, a paradigm that integrates advanced technologies such as the Internet of Things (IoT), Cyber-Physical Systems (CPS), Artificial Intelligence (AI), and Big Data analytics into production systems [1]. This shift aims to meet the growing demand for mass customisation, improve operational efficiency, and reduce time-to-market while enabling industries to respond dynamically to supply chain disruptions and market volatility [2].

**Fig. 1:**
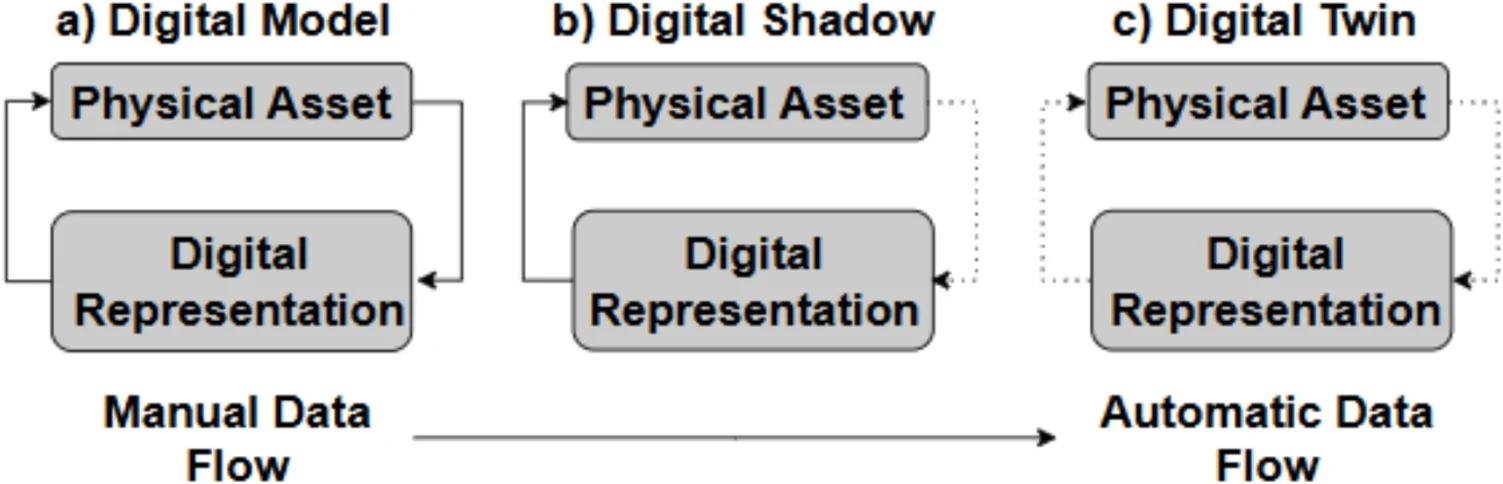
Digital transformation of a physical asset from a Digital Model (**a**), a complete manual data flow with no communication between either entity (straight lines), to a Digital Shadow (**b**), a digital representation updated to reflect the physical asset (dotted line reflects an established connection that allows communication from the asset to the digital representation, to a Digital Twin (**c**), a complete Automatic flow of data, where both entities can communicate and exchange data (dotted lines)

A key enabler of Industry 4.0 is the Digital Twin (DT), a digital representation of an asset, production line, or system that facilitates real-time monitoring, simulations, operational control, and predictive analytics by enabling bidirectional communication between the physical and digital realms [3, 4]. A fundamental distinction between DTs and DSs lies in their data exchange and control mechanisms. DSs primarily function as data mirrors, passively collecting real-time information from physical systems but lacking the ability to send control feedback [5]. In contrast, DTs actively interact with their physical counterparts, enabling real-time adaptability, prescriptive analytics, and autonomous decision-making, which are particularly useful for optimising workflows and preventing system failures [6], and are particularly advantageous in robotic process automation [2]. DTs have demonstrated significant potential in energy management systems, particularly in energy-intensive industries, by allowing accurate predictions of energy use and optimised scheduling and control strategies to improve efficiency [7]. For example, Billey and Wuest [8] developed an energy DT for a smart manufacturing system, allowing real-time monitoring and optimisation of energy usage, leading to improved energy efficiency and reduced operational costs. Similarly, Li et al. [9] proposed a system-level energy-efficient DT framework for runtime control of batch manufacturing processes, demonstrating significant energy savings through optimised scheduling and control strategies. Additionally, DTs have been applied to predictive maintenance and resource allocation, enabling systems to adapt dynamically to changing conditions [7]. Figure 1 illustrates the different ways of digitally representing an asset: (a) Digital Model, (b) Digital Shadow, and (c) Digital Twin [10].

The Asset Administration Shell (AAS) has emerged as a cornerstone of Industry 4.0 and a structured way of implementing DTs, providing a standardised framework for representing digital assets throughout their lifecycle [11]. Initially developed from the German Industrie 4.0 initiative [12, 13], AAS offers a modular metamodel that encapsulates the functional, operational, and lifecycle data of an asset into submodels, ensuring semantic interoperability and seamless integration across diverse manufacturing systems [14]. By structuring assets into well-defined submodels, AAS facilitates efficient data management throughout the value chain, from design to decommissioning, thereby eliminating information silos and fostering collaboration between otherwise incompatible systems. The implementation of AAS has demonstrated versatility in multiple industrial domains, including predictive maintenance [15], adaptive production scheduling [16], and energy optimisation [17, 18]. In energy management, predictive analytics within AAS frameworks can forecast excessive energy consumption and implement mitigation strategies, leading to measurable cost savings and environmental benefits. Similarly, in production scheduling, AAS implementations dynamically allocate manufacturing resources based on real-time data regarding resource availability and operational constraints, optimising efficiency, and reducing downtime. Recent innovations have introduced AAS frameworks, significantly expanding the capabilities of traditional AAS implementations by integrating machine learning, advanced analytics, and IoT technologies [18]. These frameworks enable assets to autonomously negotiate and optimise tasks, making real-time decisions within interconnected manufacturing ecosystems. Integration of workflow management systems and AI-driven decision-making has further improved self-optimisation capabilities, enabling smart factories to reduce delays and increase performance [19]. For instance, agent-based AAS frameworks empower autonomous components to coordinate tasks, improving responsiveness and operational efficiency.

Beyond individual factories, AAS improves value chains by improving transparency, collaboration, and supply chain resilience [20, 21]. By providing a unified digital representation of assets and their relationships, AAS facilitates collaborative decision-making among stakeholders, mitigating disruptions caused by events such as the COVID-19 pandemic and geopolitical conflicts [22]. Real-time supply chain monitoring and contingency planning enabled by AAS frameworks ensure operational continuity during unforeseen interruptions. AAS integrates real-time production data with machine learning, enabling early defect detection and predictive quality control [23, 24]. This approach is critical for zero-defect manufacturing, particularly in the automotive and aerospace industries. Additionally, intelligent inspection tools dynamically adjust parameters based on real-time data, optimising accuracy and efficiency [25]. AAS optimises energy consumption in energy-intensive industries using predictive models and advanced analytics [17, 26]. Energy management systems leverage AAS to forecast energy needs in different production scenarios, enabling proactive adjustments that reduce costs and environmental impact. AAS simplifies integration efforts by providing a structured representation of diverse assets, facilitating interoperability in multi-vendor environments [27]. Integration of AAS with data-sharing frameworks, such as Gaia-X [28] and International Data Spaces (IDS), has strengthened secure and interoperable data exchange, addressing concerns about data sovereignty in global manufacturing ecosystems [29].

Despite significant advancements, AAS implementations continue to face major challenges, particularly in interoperability, scalability, legacy system integration, real-time communication, and data security [10]. The heterogeneity of the data structures and dependencies of the proprietary system makes seamless cross-platform communication difficult, limiting the scalability of AAS in fully autonomous Industry 4.0 environments [11]. Although standardisation efforts such as the Plattform Industrie 4.0 initiative have made progress, practical implementations still lack uniformity, leading to fragmented approaches that reduce interoperability [10]. The reliance on vendor-specific technologies further hinders widespread adoption, forcing industries to create custom integration layers to bridge gaps between different AAS implementations [27]. Moreover, integrating AAS with legacy industrial infrastructures remains a significant challenge, as older systems lack the flexibility to interact with modern digital architectures, requiring costly middleware solutions and adaptable gateway architectures to bridge this gap [27, 30]. Furthermore, ensuring low-latency, bidirectional communication between multiple industrial assets is a persistent challenge, as real-time data synchronisation and consistency must be maintained to prevent discrepancies between AAS instances and their corresponding physical components [30]. Data security is an additional concern, particularly since AAS frameworks handle increasing volumes of sensitive industrial data. The concept of industrial dataspaces is gaining traction, providing secure, structured data-sharing solutions that address concerns about data sovereignty and access control [31]. Scalability and real-world deployment challenges also persist, as many existing AAS frameworks have been tested only in controlled environments and have limited practical implementation in live industrial settings. Addressing these issues will require more robust standardisation efforts, hybrid communication protocols, enhanced security architectures, and real-world validation through industrial asset deployments [11].

This study presents a framework designed to enhance the scalability and interoperability of AAS systems by enabling real-time data exchange, multi-asset synchronisation, and orchestration. The framework integrates Message Queuing Telemetry Transport (MQTT)-based communication and REpresentational State Transfer Application Programming Interface (REST APIs), establishing bidirectional communication mechanisms that improve data consistency and cross-platform accessibility. A case study demonstrates its scalability by automating the creation of three standardised AASs for two KUKA R600 robots and a KUKA TITAN 1000, where a single step ensures uniform AAS generation, stores collected data in a database, and registers AAS types in a central registry, creating a centrally secure dataspace. Another case study involving the coordination of a MiR AGV and a KUKA robot further highlights the practical benefits of this approach, demonstrating its potential to advance Industry 4.0 ecosystems towards greater autonomy and resilience. The methodology aims at facilitating the transition from digital representations to more advanced DTs while supporting interoperability and automation in smart manufacturing systems. By structuring AAS models, assets are consistently represented, ensuring cross-platform compatibility and seamless integration into digital shopfloors [21]. Additionally, the framework supports the integration of legacy systems, enabling existing industrial infrastructures to transition to fully connected, adaptive manufacturing environments without requiring complete system overhauls.

The remainder of this paper is organised as follows: Section 2 details the methodology, beginning with the general framework, creating an AAS, and the required server and registry additions to support the storage and query process, serving as a dataspace for the framework. Section 3 presents use cases that demonstrate the implementation of an AAS ecosystem in two real-world scenarios. Section 4 discusses challenges, potential improvements, and future work. Finally, Section 5 covers key findings, possible future research directions, and recommendations to improve AAS-based DTs for industrial automation.

## Methodology

This section presents the proposed methodology for a connected manufacturing environment using Asset Administration Shells as embodiments of DTs. This process involves constructing the AASs, establishing bidirectional real-time communication with the asset and other AASs, storing metadata and live data from the assets, and facilitating collaboration among multiple manufacturing assets.

### The underlying digital infrastructure

Before adopting AAS, coordinating tasks across different robots or systems often required custom integration logic, vendor-specific APIs, or centralised control programmes. These methods were typically rigid, making it difficult to adapt or scale the system when new assets were added or workflows changed. Each new integration required additional development time and created tight coupling between systems, increasing complexity and reducing maintainability. In contrast, the AAS provides a modular, standardised way to describe and access the capabilities and status of each asset. Instead of writing direct communication logic between assets, each robot interacts with the AAS layer, which abstracts away the technical differences. This allows different vendors, platforms, and programming environments to cooperate without rewriting code or reconfiguring the network.

**Fig. 2:**
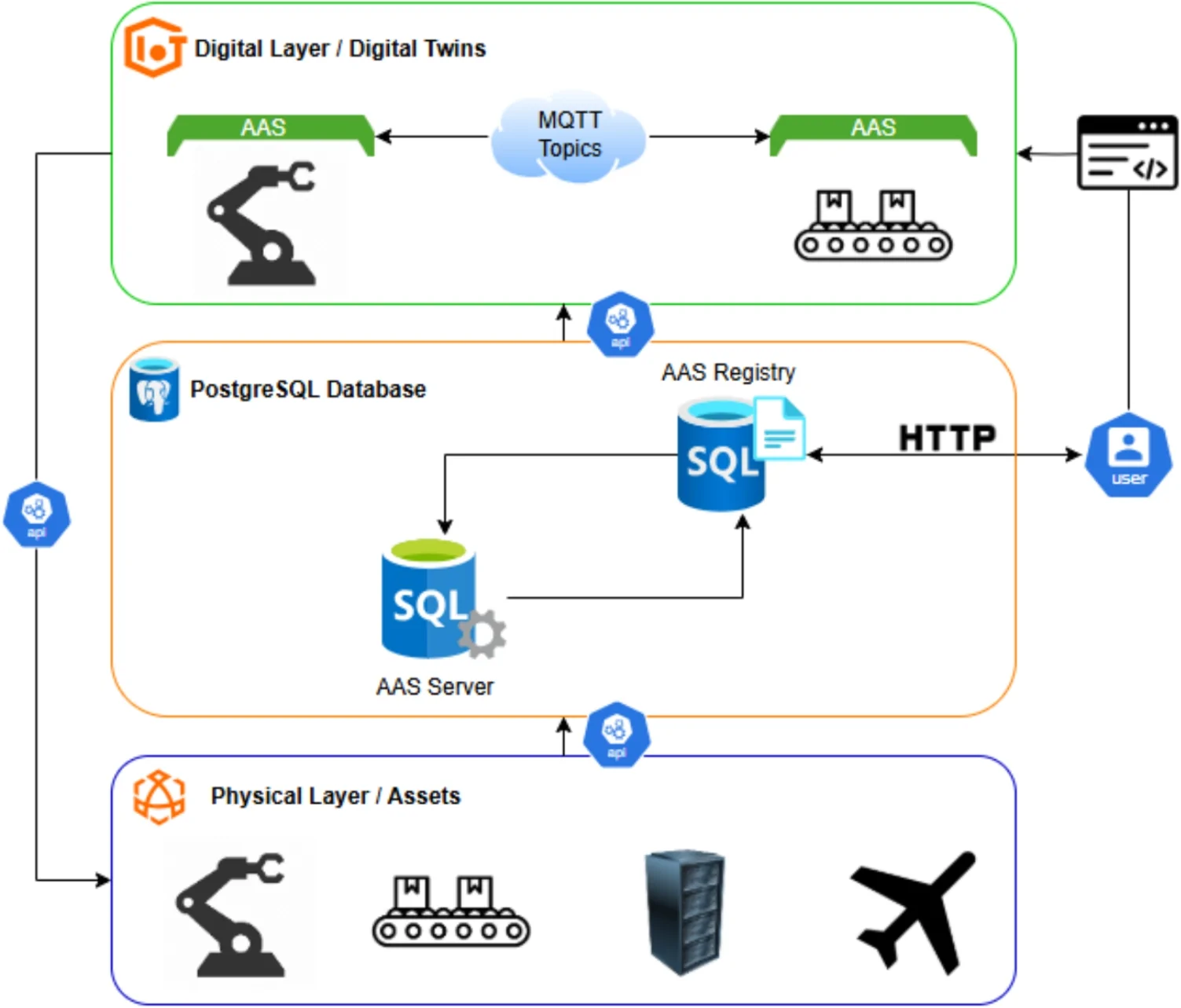
Graphical abstract of the implemented framework, where data is collected from the physical layer/assets and used to create the respective AASs. Data exchange between the AAS and its asset follows a REST API protocol, while communication between the AASs adheres to the MQTT protocol

The proposed framework, illustrated in Fig. 2, provides a structured, interoperable approach for integrating AASs into an industrial environment. It comprises three interconnected layers: the Physical Layer, the Database Layer, and the Digital Layer. Each plays a vital role in maintaining system-wide synchronisation and interoperability.

The Physical Layer encompasses industrial assets such as robotic systems, conveyor mechanisms, and other manufacturing components that generate operational data. This data is collected through REST API requests and transmitted to the PostgreSQL Database Layer. It serves as the framework’s dataspace, where the AAS Server processes, structures, and stores it in a relational database. The AAS Registry is a centralised entity for managing AAS instances, assigning unique identifiers to each registered asset, and ensuring discoverability through structured metadata and HTTP-based protocol endpoints. The Digital Layer embodies the AAS-driven DT architecture, in which a corresponding AAS instance models each asset. These instances encapsulate multiple submodels, categorising asset-specific data such as telemetry, control variables, and execution states. Within the Digital Layer, an MQTT broker establishes a synchronised data exchange between multiple AASs and their instances. Each AAS submodel is published on an MQTT topic, enabling other AASs and their instances to subscribe to data updates from specific submodels listening to topics of interest. This leads to dynamic adjustments based on the subscribed data. This bidirectional communication ensures that interconnected assets remain synchronised without requiring external intervention.

Additionally, a key aspect of the framework’s implementation is the systematic retrieval and organisation of real-time operational data. When a change is detected in a relevant submodel element, an automated update process is initiated, ensuring immediate orchestrated changes across connected assets. Furthermore, all AAS data is persistently stored within the AAS server, facilitating historical analysis, structured querying, and long-term accessibility for analytics and optimisation. By integrating these layers, the framework provides a solid foundation for industrial automation, transforming AAS instances from passive data repositories into active orchestrators of system-wide interactions.

### Creation of Asset Administration Shells (AAS)

**Fig. 3:**
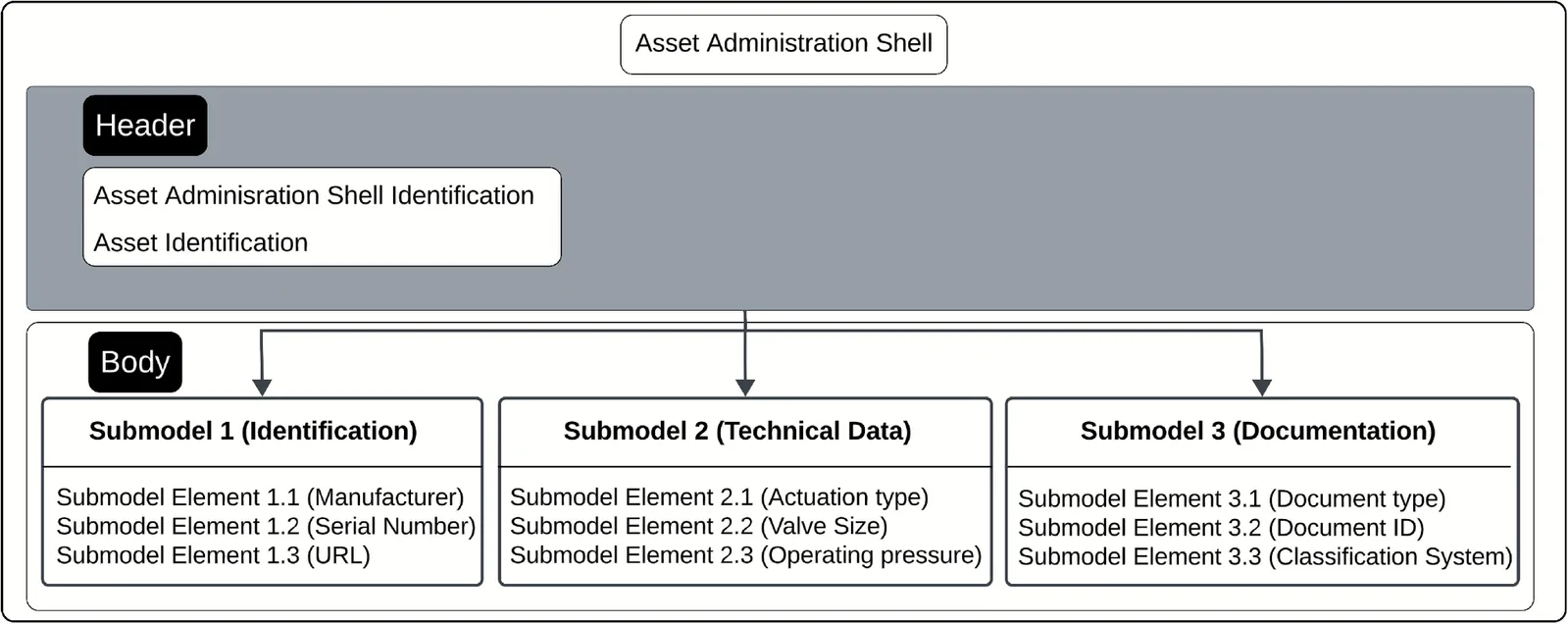
Illustration of the structure of an Asset Administration Shell [32]

Each AAS instance consists of a header and a body (see Fig. 3). The header serves as the unique identifier for an asset within a distributed system, ensuring structured interactions and traceability across interconnected networks. It includes metadata such as the AAS ID, asset ID, and asset name, which collectively define the asset and its corresponding digital twin. This standardisation guarantees that assets can be uniquely recognised and accessed within an industrial network, preventing data conflicts and enhancing integration with existing and future digital infrastructures [23]. The concepts of type and kind are central to asset identification, where type refers to the general category of an asset, such as a robotic arm model. In contrast, the kind represents a specific instance of that asset [14]. This distinction ensures that instance-specific data, including operational parameters, maintenance history, and performance metrics, can be efficiently managed without losing the generality required for large-scale industrial automation. Additionally, the header facilitates the modelling of complex asset relationships, allowing the representation of interconnected systems, such as production lines comprising multiple components, including robots, conveyors, and sensors [32].

The body of the AAS comprises multiple submodels that organise asset information into structured categories, enabling modularity, reusability, and systematic data retrieval. Each submodel represents a specific characteristic of the asset and is further segmented into submodel elements, which encapsulate individual data points or functional attributes [13]. The identification submodel contains essential details such as the manufacturer, serial number, and reference URLs [14]. The technical data submodel defines key operational parameters, including the type of actuation, material properties, and environmental conditions under which the asset functions [11]. These submodels collectively provide a comprehensive description of the asset’s capabilities and operational constraints, supporting informed decision-making in automated manufacturing environments [23]. In addition to static asset specifications, the AAS incorporates submodels that capture dynamic and real-time asset behaviour. For example, the operational data submodel stores real-time performance metrics such as velocity, torque, energy consumption, and temperature variations, enabling continuous monitoring of asset performance [13]. The navigation submodel is particularly relevant for mobile assets, as it contains localisation data, movement constraints, and the trajectory-planning information necessary for autonomous operations [14]. Similarly, the mission and process data submodel ensures that assigned tasks, execution states, and workflow interactions between different AAS instances are accurately represented, supporting synchronised and collaborative manufacturing processes [32]. The submodel elements within each submodel provide granular detail, ensuring that every data component in the AAS adheres to a consistent and machine-readable format. These elements are linked to standardised ontologies and shared semantic models, enabling seamless data interpretation across multiple platforms [32]. This approach improves interoperability and enables efficient querying, retrieval, and integration into larger digital ecosystems [14].

The structured, modular nature of the AAS submodels supports event-driven interactions, enabling assets to autonomously trigger responses based on predefined conditions. For instance, an operational threshold defined within the lifecycle submodel can initiate a maintenance request when specific wear indicators exceed a predetermined limit [31]. This capability ensures that manufacturing systems remain responsive and adaptive, minimising unplanned downtime and optimising asset performance [11]. Furthermore, integrating external data sources, including CAD models, IoT sensor feeds, and enterprise resource planning (ERP) systems, enables a comprehensive digital representation that bridges the gap between static specifications and dynamic operational data [32].

By providing a consistent, extensible framework for asset representation, the AAS lays the foundation for achieving Industry 4.0 objectives in automation, interoperability, and data-driven decision-making [14]. The structured approach to identification, metadata organisation, and submodel categorisation allows for incremental integration with legacy systems while maintaining the flexibility required for future advancements in industrial automation [13]. Seamlessly incorporating static and dynamic asset data ensures that digital twins remain accurate representations of their physical counterparts, supporting predictive maintenance, process optimisation, and real-time decision support in interconnected industrial environments [31].

### Implementing a bidirectional data exchange mechanism

**Fig. 4:**
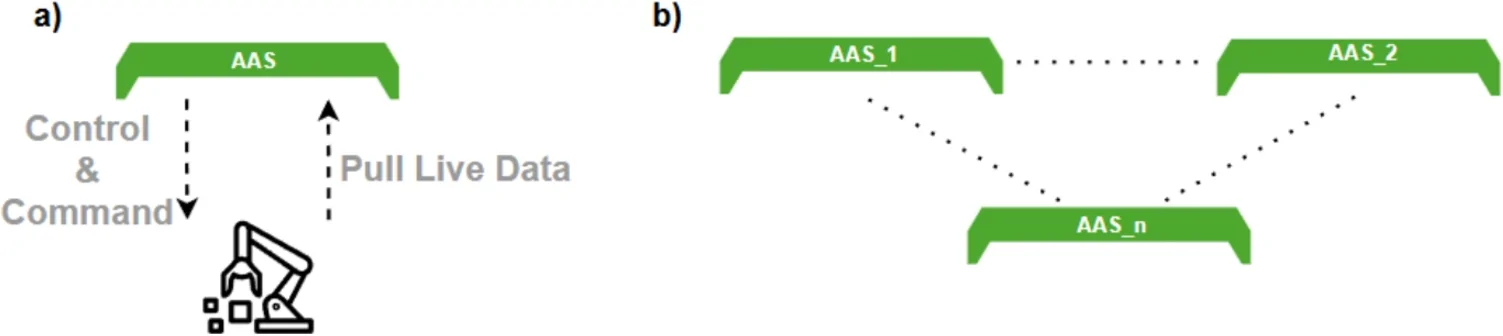
(**a**) Bidirectional data exchange between the physical asset and its AAS, and (**b**) Direct communication and data exchange between multiple AASs of different assets

Figure 4 illustrates communication between an AAS and its corresponding asset through a continuous data exchange loop. The incoming data flow ensures that the AAS remains synchronised with the physical asset by continuously retrieving real-time operational data via standard Application Programming Interfaces (APIs). This data includes performance metrics, diagnostic logs, and system status updates, providing a real-time representation of the asset’s operational state. Similarly, the outgoing data flow allows the AAS to send control commands to the asset, allowing dynamic system reconfiguration, parameter adjustments, and real-time workflow modifications. This bidirectional communication loop is essential for predictive analytics, anomaly detection, and process optimisation, ensuring that real-time feedback and adaptive control mechanisms enhance efficiency, responsiveness, and system resilience.

Beyond direct communication between assets and AAS, interoperability between multiple instances of AAS must be established to enable collaborative decision-making and optimise distributed systems [33]. This can be achieved using Message Queueing Telemetry Transport (MQTT), an event-driven messaging protocol designed for low-latency, high-frequency data exchange. MQTT enables AAS instances to subscribe to relevant data streams, ensuring that only contextually significant information is exchanged, reducing bandwidth consumption and minimising communication latency. This model supports autonomous coordination of industrial processes, particularly within automated production lines and synchronised manufacturing workflows.

**Fig. 5:**
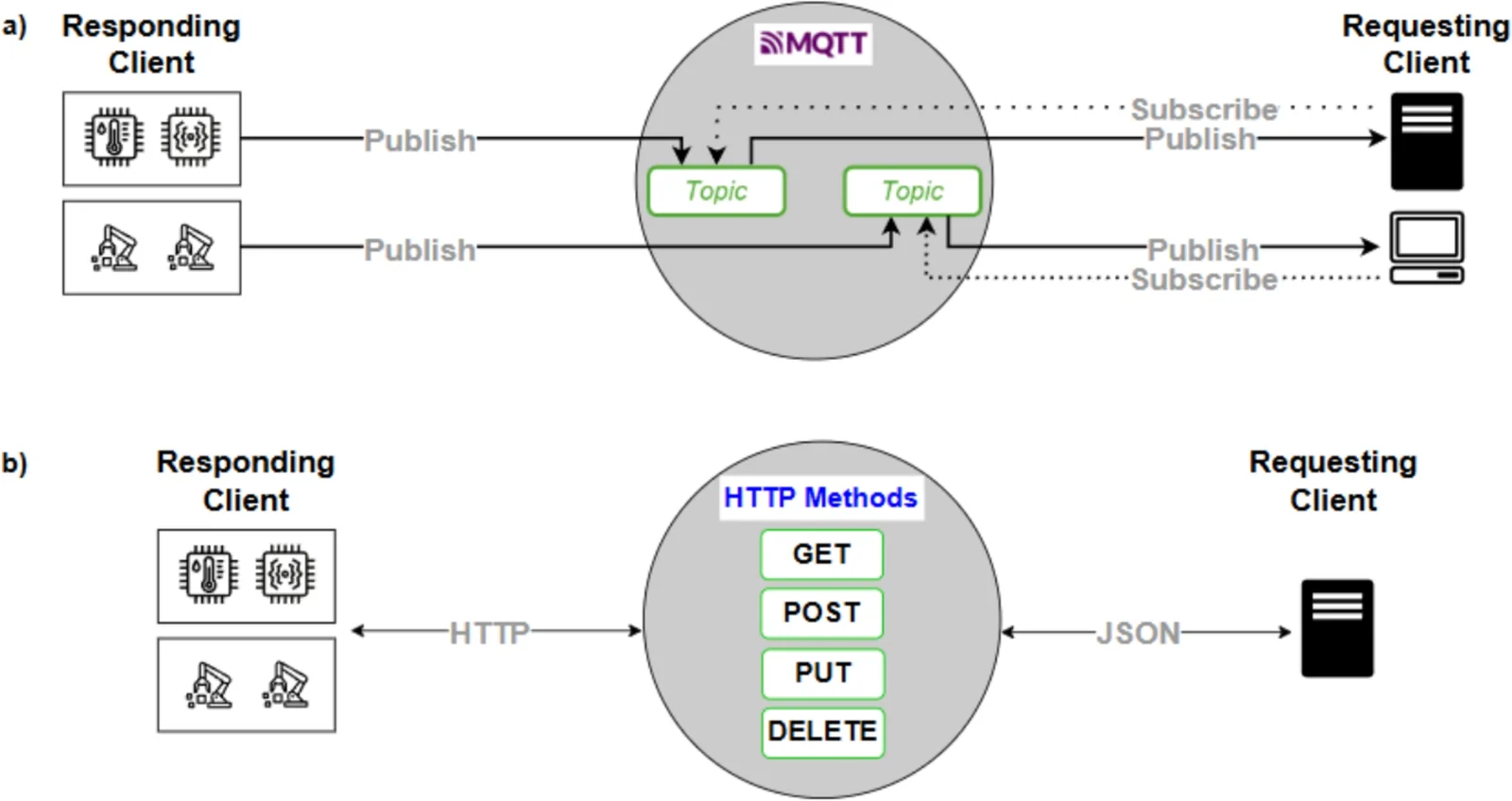
(**a**) The Publish/Subscribe data exchange mechanism followed in the MQTT protocol, and (**b**) Data transfer request protocol for REST API methods

The proposed framework integrates MQTT with REST APIs, combining the strengths of both protocols to provide a flexible and scalable data exchange mechanism. As illustrated in Fig. 5, MQTT follows a publish/subscribe model, where AAS clients publish data to specific topics hosted on an MQTT message broker. Other clients subscribe to these topics, receiving only the relevant data, enhancing system efficiency and scalability. This event-driven approach is convenient for real-time applications such as predictive analytics, live monitoring, and synchronised asset coordination, ensuring that industrial systems remain adaptive and responsive to changing conditions [34].

While MQTT provides high-speed, low-latency communication, REST APIs follow a request-response model using standard HTTP methods (GET, POST, PUT, DELETE) for structured data retrieval and modification. REST APIs exchange data in JSON format, ensuring platform neutrality and structured interoperability across enterprise IT systems. This method is well-suited for structured data management, enabling controlled access to asset information while ensuring compatibility with existing data storage frameworks. Hence, it is used to retrieve data from assets.

Other messaging services include the Constrained Application Protocol (CoAP), the Advanced Message Queuing Protocol (AMQP), and the Data Distribution Service (DDS), each offering distinct capabilities but with limitations compared to MQTT for AAS communication. CoAP, while optimised for lightweight machine-to-machine communication, is primarily suitable for constrained networks and lacks the advanced publish/subscribe mechanisms required for scalable event-driven industrial applications [35]. AMQP, a robust queueing protocol designed for enterprise-level messaging, ensures reliable delivery but introduces higher overhead and complexity, making it less efficient for real-time data exchange in resource-constrained environments [36]. DDS, a high-performance data-centric protocol, provides granular quality-of-service (QoS) settings and real-time data distribution; however, its complexity and resource requirements make it less suitable for applications that require lightweight, efficient messaging. In contrast, MQTT’s minimal bandwidth requirements, low latency, and efficient publish/subscribe architecture make it particularly well-suited for AAS interactions, enabling seamless data exchange and synchronised asset coordination across industrial systems [37]. By integrating MQTT with REST APIs, this framework leverages the advantages of both real-time event-driven messaging and structured data management, ensuring a scalable, interoperable, and adaptive communication model for Industry 4.0 environments. Additionally, this hybrid approach supports flexible, efficient, and secure industrial data management, facilitating seamless interactions between AAS instances, physical assets, and enterprise applications. Thus, it ensures that Industry 4.0 ecosystems remain highly adaptable and robust.

### Data storage, query, and log

**Fig. 6:**
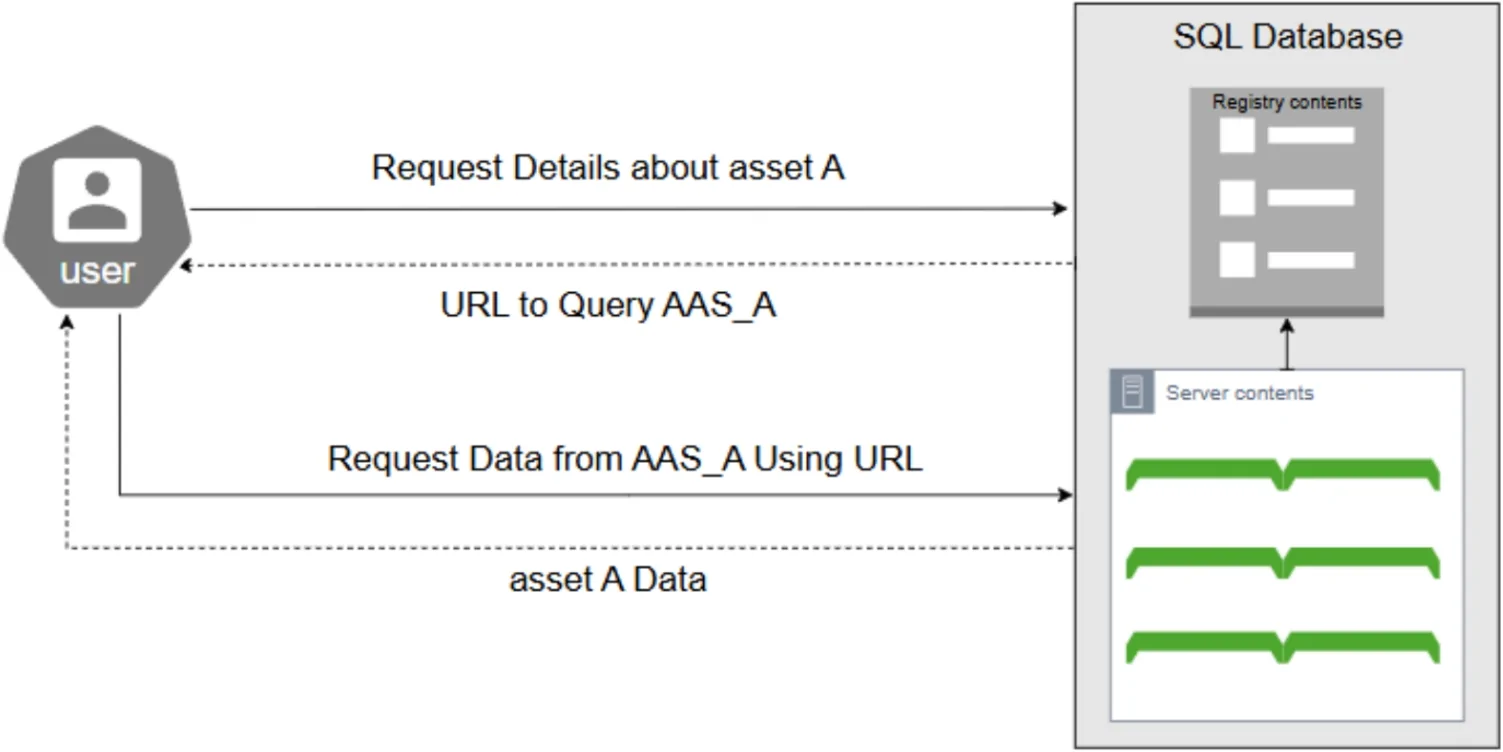
Framework followed for User/Client to request data from a specific asset. First, the endpoint URL of the asset is identified from the AAS registry and used to query data from the AAS server

Implementing an AAS-based digital manufacturing system requires a structured, scalable dataspace that supports efficient storage and querying, real-time data updates, and the log of newly created entities [15]. This framework includes a database running on a server to store the vast amounts of data generated by all the assets, and a registry to identify and log these assets and explain how to access their data. Figure 6 illustrates how a database of AAS Server and AAS Registry can be used to connect a user to an asset’s data.

A common challenge is ensuring data consistency across decentralised AAS implementations. As manufacturing systems increasingly rely on multi-vendor solutions, query mechanisms must conform to standardised data formats [31]. Semantic data structuring using linked data models and ontology-based frameworks ensures that queries across AAS instances yield consistent, interoperable results. By addressing these considerations, AAS-based storage and query systems can enhance efficiency, facilitate seamless integration, and support large-scale industrial automation [38].

#### AAS registry

The AAS registry is a centralised directory that maintains a structured list of registered AAS instances, their corresponding endpoint URLs, and metadata descriptors. This registry functions similarly to a networked directory service, enabling applications and other AAS instances to dynamically discover, register, and communicate with each other. The registry facilitates traceability, discoverability, and standardised access across heterogeneous DT ecosystems by implementing unique asset identifiers. Each entry in the registry includes a unique identifier (UID) for the asset and the URL of the AAS endpoint, thus facilitating direct access to asset information.

A significant advantage of the AAS registry is its ability to enable dynamic system updates. In conventional asset management, tracking changes in an asset’s lifecycle—such as deployment, maintenance, or decommissioning—can often be challenging [3]. However, with an event-driven registry architecture, newly instantiated AAS instances can be automatically registered, while outdated instances are dynamically removed to prevent network inconsistencies. This guarantees that only active and relevant DTs are available for queries, thereby reducing computational overhead and enhancing system performance.

Furthermore, AAS registries also improve cross-platform interoperability by providing a lookup mechanism compatible with Industry 4.0 data exchange standards. By utilising semantic web technologies and ontology-based classification, registries can facilitate cross-enterprise collaboration, allowing AAS instances from different organisations to securely share and retrieve asset-related data within decentralised industrial ecosystems [2]. Ultimately, the AAS registry serves as the foundation for scalable and autonomous industrial automation. It provides structured, real-time access to interconnected asset networks while maintaining high levels of interoperability and security.

#### AAS server

The AAS server is the central processing unit responsible for hosting, managing, and synchronising DTs within an Industry 4.0 ecosystem. It provides a structured mechanism for interaction between cyber-physical components, facilitating real-time communication, data storage, and DT lifecycle management [39]. The database containing the AAS server is designed to handle large volumes of asset data, ensuring scalability and robustness in industrial environments. The AAS server supports multiple communication protocols, including MQTT, OPC UA, and REST APIs. Implementations such as Eclipse BaSyx and AASX servers provide middleware that bridges operational technology (OT) and information technology (IT) systems, ensuring seamless integration with existing industrial networks [40]. Beyond data storage, AAS servers also manage access control and security, ensuring that only authorised entities can query or modify asset data. Role-based access control mechanisms allow manufacturers to enforce detailed permissions, restricting asset visibility and control based on user privileges and organisational policies. In large-scale industrial applications where multiple DTs interact in real time, AAS servers can implement event-driven architectures, enabling asynchronous data exchange via message queues and real-time event streams [30]. This improves system efficiency and ensures low-latency responses to critical operational updates.

## Implementation and use cases

### AAS creation and connection to server and registry

**Fig. 7:**
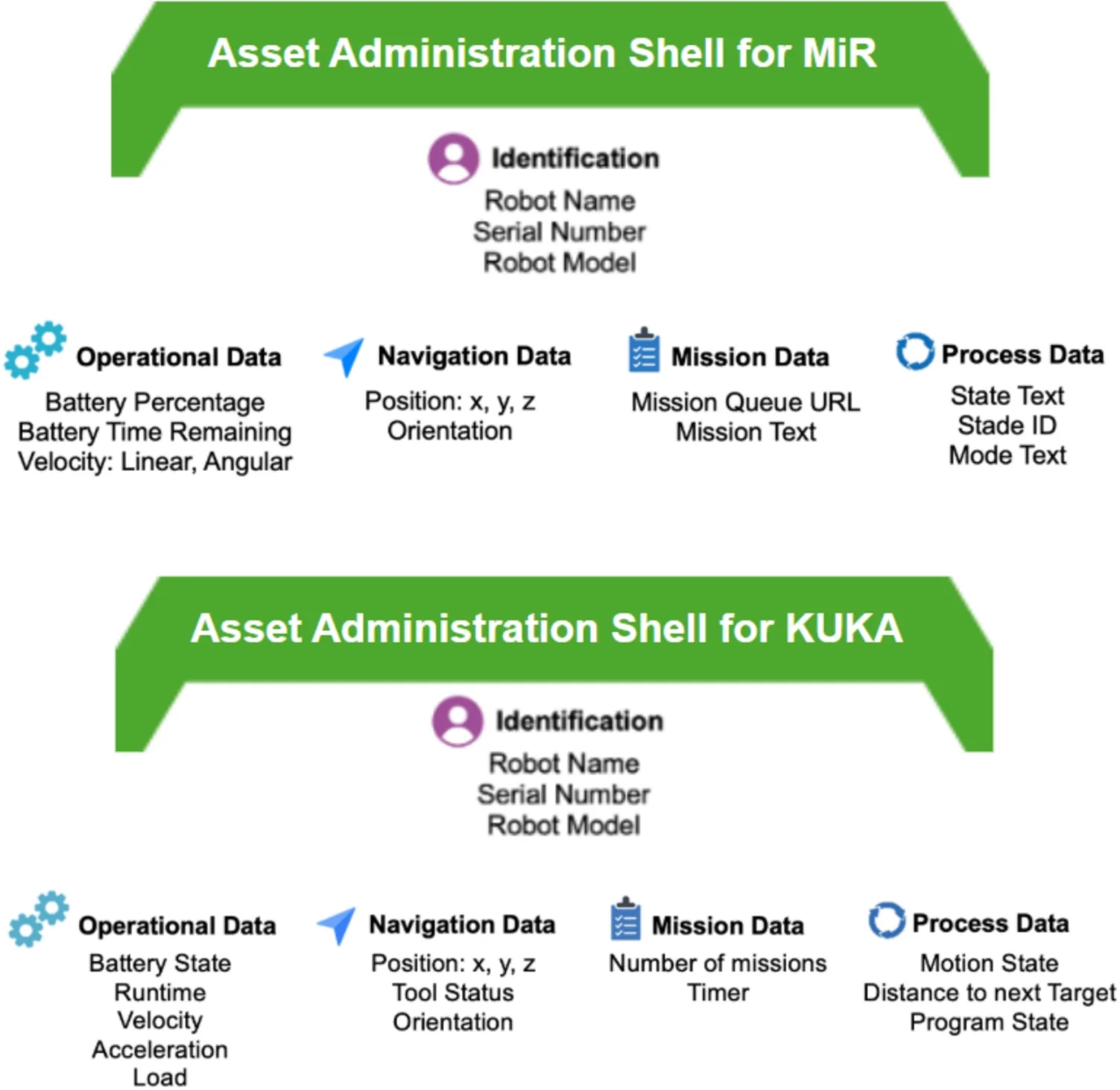
The structure of the AAS for both the MiR and KUKA robots, showing the header data used for identification and Submodel elements reflecting the asset’s data

To achieve the foundational principles of DTs, four AASs were developed for two KUKA KR4 R600 educational robots, a KUKA KR1000 TITAN industrial robot, and a MiR 250 autonomous mobile robot. These ensure that each system entity has a structured digital counterpart, serving as the primary assets and AASs for the two implemented use cases. Figure 7 shows the structure of an AAS for a MiR and a KUKA robot. The contents depict the AAS instances for industrial assets: MiR autonomous mobile robot and KUKA robots. These AAS instances provide a standardised, machine-readable format for asset information, enabling seamless data exchange and automated control.

From Fig. 7, the AAS consists of a Header section that serves as an *Identification* section; it contains information such as Robot Name, Robot Model, and Serial Number. The second section is the Body, which includes submodels representing its *operational state*, *navigation details*, *Mission description*, and *configuration settings*.

For the MiR, the *OperationalData* submodel includes parameters such as battery percentage, remaining battery time, current velocity, and state text. The *Navigation* submodel records information such as distance to the next target, current mission status, and movement timestamp, ensuring continuous tracking of robot activities. Additionally, the *ConfigurationAndSettings* (process) submodel captures safety and operational configurations, such as State Text and Mode Text.

The AAS of the KUKA robots follows a similar structured approach, but with submodels specifically tailored to an industrial robotic arm. The *Operational Data* submodel includes acceleration parameters, battery state, robot runtime, and velocity, providing information on the robot’s dynamic behaviour. The *Navigation Data* submodel contains position-related attributes, such as orientation, end-effector position, and tool status, ensuring precise tracking of the robot’s movements. Additionally, the *Mission Data* submodel maintains the number of assigned missions and an active mission timer, which reflect the parameters controlling the programme the robot is running. In contrast, the *Process Data* submodel includes details like motion state and programme execution status.

The created AAS instances were registered in the registry and stored in the PostgreSQL database. The registration process creates an instance of the AAS in the registry and assigns a URL endpoint for structured querying of the asset data. This registry enables real-time asset discovery and guidance for querying a specific asset, addressing a limitation observed in earlier static AAS models. The created server database consisted of the following four tables:

Table 1 shows the AAS server in the database, which consists of four other tables. First, the AAS table includes the asset ID for identification, the asset’s serial number, the asset name, a description of what this registry represents, and the creation time. The submodel table contains a generated ID for each submodel, the AAS to which it belongs, the submodel name, and the creation time. Similarly, the submodel element table details the data tags collected from the assets along with their respective IDs. These three tables contain metadata on the AASs developed; the remaining table, the submodel element history, stores the actual data from the assets. The history of submodel elements records the values for the data tags and the IDs of the corresponding submodels and assets. Table 2 shows the AASs developed and their identification:

**Table 1:** Four tables formed to structure the Server database where all asset data is stored

Schema	Name	Type	Owner
Public	aas	table	postgres
Public	submodel	table	postgres
Public	submodel element	table	postgres
Public	submodel element history	table	postgres

**Table 2:** AAS table showing all created asset administration shells

ID	Name	Description	Created At
8144xxx	KUKA KR4 R600	AAS for KUKA KR4 R600	yyyy-mm-dd HH:MM:SS
8144xxx	KUKA KR4 R600	AAS for KUKA KR4 R600	yyyy-mm-dd HH:MM:SS
990xxx	KUKA TITAN KR1000	AAS for KUKA TITAN KR1000	yyyy-mm-dd HH:MM:SS
20230xxx	MiR 202303742	AAS for MiR 202303742	yyyy-mm-dd HH:MM:SS

The following sections detail two specific use cases that illustrate how this architecture enables real-time robot synchronisation and event-driven task execution in a manufacturing environment.

###  Use case 1: live data exchange between two KUKA robots for synchronised operations

The first use case demonstrates the synchronisation of operational parameters between two KUKA KR4 R600 industrial robots, leveraging AAS instances and MQTT-based communication to ensure real-time consistency. Initially, AAS instances are created for both robots, each encapsulating key operational data within a structured submodel. These submodels define telemetry parameters, control variables, and execution states, ensuring comprehensive digital representations of the physical assets.

**Fig. 8:**
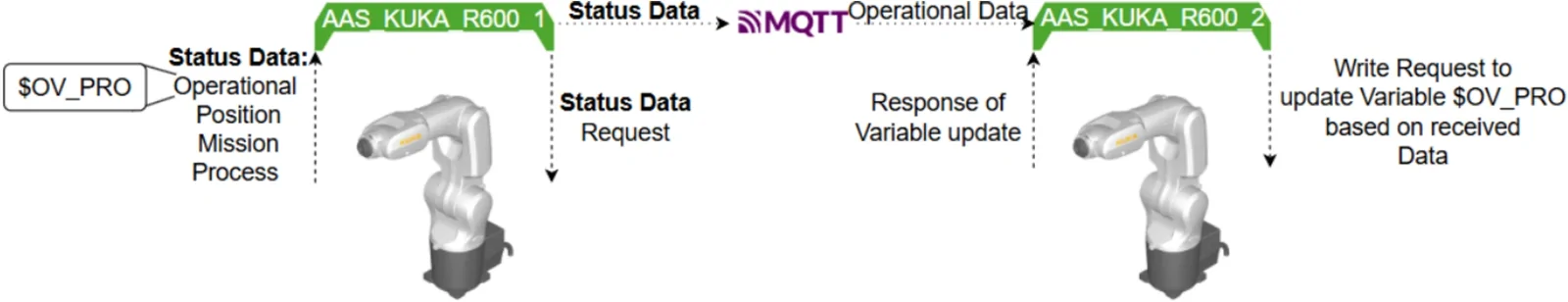
The framework for the data exchange between the two KUKA KR4 R600 robots. The second robot is subscribed to the data published by the first robot, and its AAS updates the parameters when necessary

Live data from the robots is retrieved via REST API calls, populating the corresponding submodel elements within the AAS framework. The implementation utilises MQTT for communication, with each submodel published as an independent topic. The second robot’s AAS instance is subscribed to a specific topic corresponding to a key operational parameter of the first robot. This arrangement ensures that changes to the first robot’s AAS are immediately propagated to the second robot. Figure 8 illustrates the framework and flow of data for the first use case.

For instance, during the experimental validation, the control parameter \$OV_PRO, which determines the robot’s velocity during program execution, was initially set to 50 percent. When this value was updated to 70 percent in the AAS of the first robot, the change was detected by the AAS of the second robot through the MQTT subscription. Upon detecting this update, an automated API request was sent to the control system of the second robot, synchronising its \$OV_PRO value to match that of the first. This demonstrated the capability of AAS-driven automation to maintain operational consistency across assets. This can be very useful for time-saving in production lines with multiple assets, where synchronisation is critical.

**Fig. 9:**
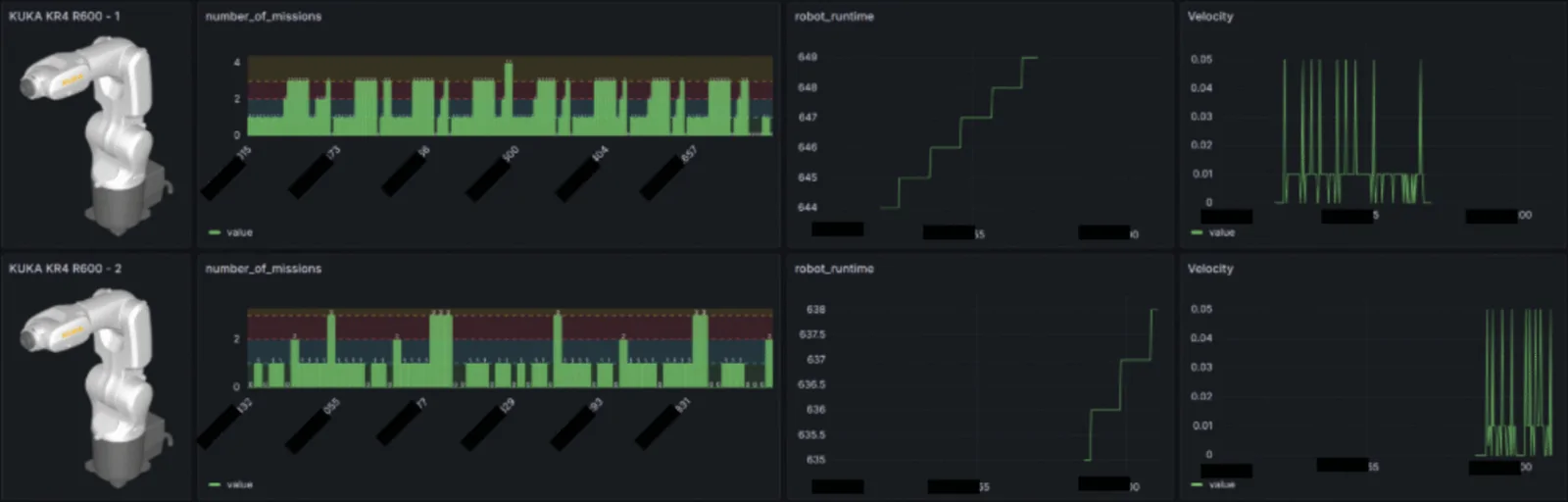
Developed dashboard using Grafana to monitor the operational parameters of the KUKA KR4 R600 robots

All data changes were recorded and stored on the AAS server, allowing the development of a real-time dashboard to monitor the robots’ operational data for data-driven decision-making, as shown in Fig. 9. Additionally, the AAS registry provided structured metadata, allowing external systems to discover and interact efficiently with the robots’ DTs. The implementation validated the feasibility of using AAS for bidirectional data exchange and synchronisation of multiple assets, reducing the need for external supervisory control while ensuring real-time consistency in robot operations.

### Use case 2: bidirectional data exchange for automated task execution

**Fig. 10:**
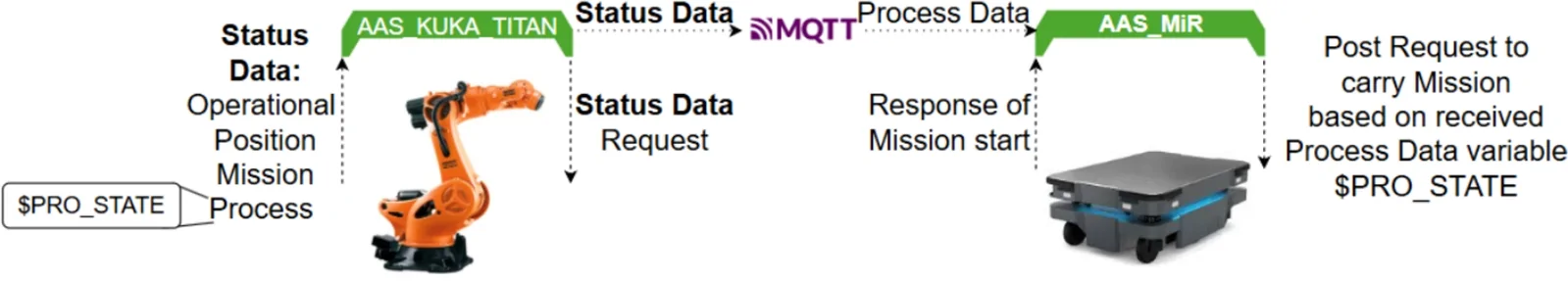
Framework of the second use case, where the MiR is subscribed to the KUKA TITAN KR1000 AAS and a mission is triggered following a specific data change

As illustrated in Fig. 10, the second use case extends the AAS-driven automation paradigm to a scenario involving a KUKA TITAN R1000 industrial robot and a MiR250 autonomous mobile robot. This implementation demonstrates how AAS instances can enable event-driven task execution in response to real-time changes in an asset’s operational state.

AAS instances were created for the KUKA TITAN R1000 and MiR250, with their data stored in submodels that correspond to their specific functionalities. REST API requests were used to continuously retrieve live data from both assets, keeping AAS instances up to date. The implementation used an event-driven control strategy using MQTT. The MiR250 was subscribed to a specific submodel element within KUKA TITAN’s AAS—specifically, the \$PRO_STATE variable, which indicates whether the robot is actively executing a task. When the KUKA robot completed its assigned task and its \$PRO_STATE switched to false, the MiR250’s AAS detected this change. In response, an automated mission execution command was triggered, instructing the MiR250 to move from its charging station to a predefined location near the KUKA TITAN.

This real-time interaction between the robots demonstrated the potential of AAS for orchestrating multi-asset automation without external human intervention. Structured AAS submodels ensured that the robots could interpret changes in each other’s states, enabling event-driven task coordination. In a large manufacturing ecosystem, this can be used to automate multiple routine steps that require an operator to trigger actions across multiple assets manually.

**Fig. 11:**
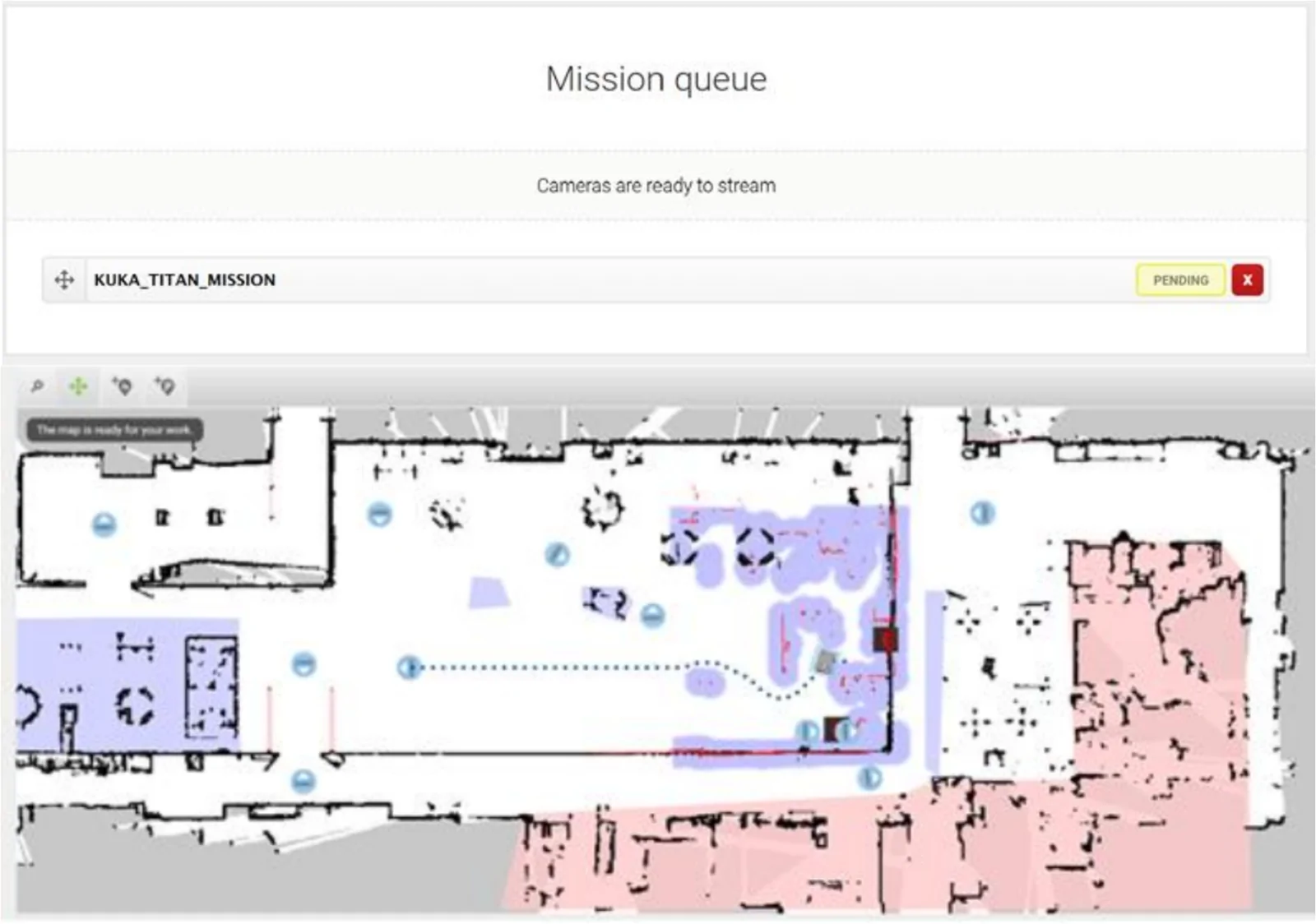
Triggered mission following KUKA KR1000 robot data change from the MiR’s UI

**Fig. 12:**
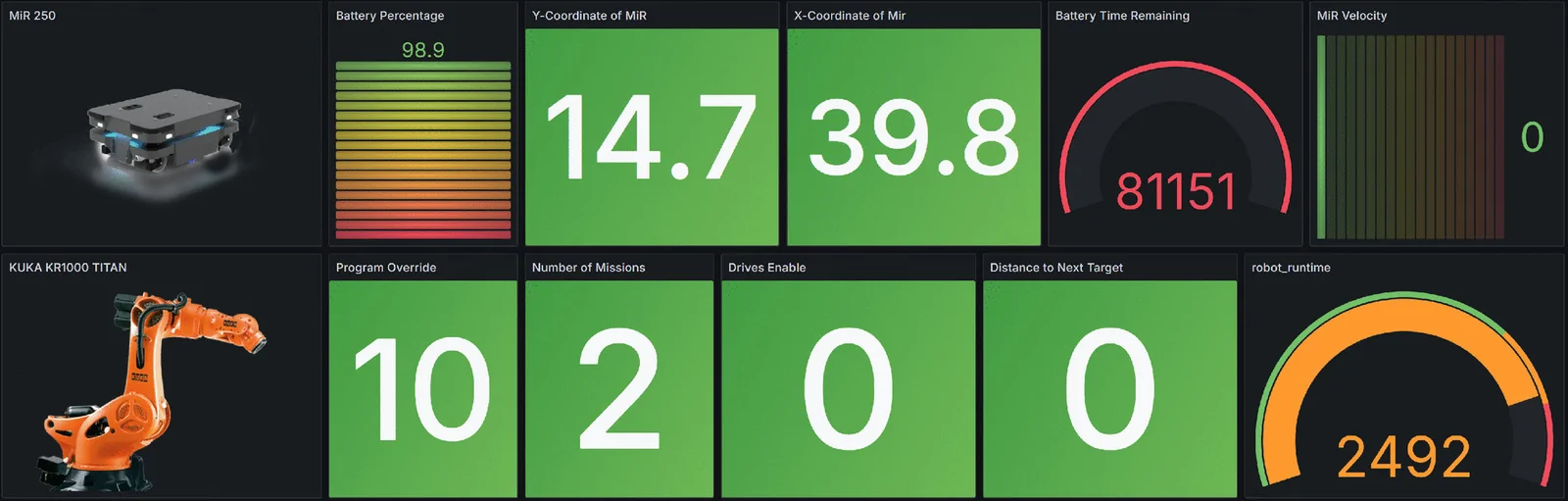
Developed dashboard to monitor the operational parameters of the MiR250 and KUKA KR1000 robots

As in the first use case, all data transactions were stored in the AAS server, ensuring traceability and enabling historical analysis. The AAS registry provided metadata about each asset, facilitating seamless integration with higher-level control systems. Figure 11 illustrates the execution of “KUKA_TITAN_MISSION”, triggered by the change in the data of the KUKA KR1000 robot. The live dashboard created from the MiR and KUKA KR1000 data is shown in Fig. 12.

### Measure of interoperability across use cases

As demonstrated in our use cases, AAS-based coordination enabled synchronising operations or triggering tasks without human intervention. This flexibility is essential for scaling to larger systems where manual integration becomes impractical and interoperability becomes challenging. Therefore, this study evaluated interoperability by assessing the successful coordination of heterogeneous assets through only the AAS interface and supported communication protocols (REST and MQTT). The KUKA robot and the MiR robot differ significantly in vendor architecture, programming environments, and supported interfaces. The two systems were able to exchange real-time status information, react to each other’s state changes, and coordinate actions through AAS without requiring custom APIs or middleware, demonstrating practical interoperability.

To validate that data published from the AAS of one asset was consistently received and acted on by the other asset, a digital handshake mechanism was implemented that triggered a signal on the MiR at the start of a mission. The KUKA read this as confirmation that the MiR received and responded to the shared AAS data. This provided an additional layer of acknowledgement to validate successful coordination. The combination of protocol interoperability, standardised submodels, and validated responses through the digital handshake served as our main criteria for demonstrating interoperability in a real-world, cross-platform context. Additionally, the metrics in Table 3 were used to assess the interoperability of the system.

**Table 3:** Interoperability and system performance metrics

Metric	Value
End-to-end latency	*∼*250 ms
Message delivery success rate	100%
Interoperable task completion rate	100% (10/10)
Reusability of submodels	High

Table 3 presents the key metrics used to evaluate the interoperability and performance of the system. The average end-to-end latency is approximately 250 milliseconds, measured from the moment the KUKA robot data was read via the REST API, published to the MQTT broker, and used to trigger a mission on the MiR robot through a second REST API call. This delay was measured using timestamp logs in repeated trials. The message delivery success rate was 100%, and all published MQTT messages were received and processed correctly by the subscribing asset. Similarly, every communication event between the KUKA and MiR robots led to the correct execution of the task, as confirmed by digital handshake signalling and system logs, resulting in a 100% % completion rate of the task. In addition, the same submodel logic for mission trigger was reused across both use cases (robot-to-robot and robot-to-vehicle), demonstrating high submodel reusability and supporting the framework’s modularity and scalability.

## Discussion

The proposed framework effectively demonstrates how AASs facilitate bidirectional real-time communication and dynamic coordination among heterogeneous industrial assets. Unlike traditional passive AAS models that primarily function as static data repositories, our implementation establishes active DTs capable of influencing physical assets and interacting with other AAS instances. Two industrial use cases validated this: synchronised velocity control between KUKA KR4 R600 robots and event-driven task execution involving a KUKA TITAN and MiR250. These scenarios highlight the framework’s ability to reduce reliance on centralised control systems by embedding logic directly within asset-level interactions. A key achievement lies in the structured integration of REST APIs and MQTT protocols to meet distinct communication requirements. As illustrated in Use Case 1, REST APIs ensured reliable retrieval of operational parameters (e.g., \$OV_PRO) for submodel updates. At the same time, MQTT’s publish-subscribe model enabled the instantaneous propagation of parameter changes among robots. This dual-protocol approach overcomes limitations observed in prior works that relied solely on HTTP-based methods. Grafana dashboards (Figs. 9–12) further illustrate how real-time data from multiple assets can be unified for monitoring, demonstrating the interoperability of the framework between robotic platforms. The modular AAS design, featuring standardised headers and customisable submodels, proved critical in accommodating various asset types. For instance, the MiR250’s AAS incorporated navigation-specific submodels (e.g., mission status, battery metrics), while the KUKA robots highlighted motion parameters (e.g., velocity, programme state). This modularity enabled seamless integration with legacy systems, as evidenced by the PostgreSQL-based registry that manages modern and legacy robot data through unified user identification numbers (UIDs). Additionally, this approach enabled automated mission triggers on the MiR asset based on external factors. Furthermore, the practical usefulness of AAS in this study lies in its ability to automate and reduce human intervention. Traditionally, asset coordination would require an operator to manually trigger missions and tasks manually, often relying on predefined workflows or custom integration scripts between systems. With the AAS framework in place, asset interactions were fully automated based on real-time data; for example, the MiR robot could monitor a specific status variable in the AAS of the KUKA robot and trigger its own mission without manual input. This significantly reduces operator workload and eliminates delays between task completion and mission initiation. The AAS acts as a shared, standardised layer that allows assets to communicate through meaningful digital representations rather than low-level APIs or proprietary protocols. This demonstrates how AAS not only facilitates communication but also transforms disconnected operations into coordinated, intelligent workflows.

While the framework achieved its objectives, several challenges emerged. First, latency fluctuations were observed during peak MQTT message throughput, particularly when multiple AAS instances subscribed to the same topic (e.g., \$PRO_STATE in Use Case 2). Scaling to more extensive networks with hundreds of assets may require edge computing strategies [29] to mitigate processing bottlenecks. In our current implementation, we use a centralised AAS server and a single MQTT broker to manage communication and coordination between physical assets. Although this approach worked efficiently for our small-scale use cases, in a larger system with many robots or devices, relying on a single server could introduce communication delays or create a bottleneck. As more assets exchange data more frequently, the time it takes to send, process, and respond to messages may increase, especially when everything goes through a single central point. To reduce this latency and improve system responsiveness, we propose enhancing the framework by adding edge computing capabilities. In this improved setup, each robot or asset would run its own local AAS instance (at the “edge”), allowing it to process data, make decisions, and communicate with nearby assets without going through the central server every time. Only important updates or logs would be sent to the central server for storage or monitoring. This would reduce network load by employing a load balancer at each edge node, making the system more scalable and allowing robots to respond to changes much faster.

Subsequently, to validate that communication between robots is not only successful but also leads to actual action, we implemented a simple digital handshake mechanism between the assets. For example, in the first use case setup, the MiR robot has a digital input that is activated when it receives a command from the KUKA robot’s AAS to begin a mission. This input changes state (from 0 to 1), and the KUKA robot monitors this change through a connected digital output. This means that after the KUKA robot publishes data using our framework, it waits for confirmation by listening to this digital signal. Once the KUKA robot detects that the MiR has received and acted on the message, it continues its own process. This setup confirms that data flow and execution were successful and helps avoid miscommunication or unnecessary delays in tightly coordinated tasks. Additionally, combining digital confirmation with edge AAS deployment provides a more reliable and scalable solution for real-world manufacturing environments.

Moreover, the digital handshake enabled more accurate latency measurement. By recording the time when the KUKA robot updated its AAS value (the triggering event) and comparing it to the time it detected the MiR’s input change, the total delay from sending the command to receiving confirmation is calculated. This provides a practical, measurable way to evaluate the system’s real-time performance beyond software-level logs or MQTT timestamps.

Second, security mechanisms were limited to basic API authentication and TLS encryption for MQTT. The bidirectional nature of AAS interactions increases the surface area of vulnerabilities, as demonstrated by recent attacks on IoT protocols [41]. Future implementations should integrate role-based access control at the submodel level to restrict sensitive data (e.g., mission routes, calibration parameters) while maintaining interoperability. Federated AAS registries that leverage blockchain [42] could enhance data consistency and trust in multi-stakeholder environments, but require testing beyond the lab-scale setup demonstrated here. Third, while the framework supports legacy system integration through REST APIs, proprietary protocols (e.g., KUKA’s KRL) require custom middleware for data translation. This aligns with broader interoperability challenges, underscoring the need for vendor-neutral protocol adapters in AAS implementations [11]. Finally, the current AAS server architecture assumes homogeneous network conditions, which may not hold in distributed supply chains.

Our framework advances AAS implementations in three key areas:

### Dynamic submodel interaction

Unlike static AAS models, our submodels support event-driven triggers (i.e., mission initiation on \$PRO_STATE changes), enabling autonomous asset coordination.

### Hybrid communication

Combining MQTT for real-time alerts and REST for structured queries addresses the protocol silos prevalent in single-protocol architectures.

### Legacy system adaptability

The PostgreSQL registry’s ability to map legacy asset data to standardised submodels mitigates transitional barriers common in Industry 4.0 migrations.

Future work will focus on four primary areas. First, we aim to implement edge-based AAS instances to reduce latency and reduce reliance on central servers, enabling more responsive, distributed coordination. Second, we will develop semantic submodel templates for common asset types (e.g., AGVs, CNC machines) to accelerate deployment and improve interoperability. Third, we plan to integrate machine learning models within submodels to enable predictive adjustments and optimise production performance using real-time asset data. Fourth, as the system scales beyond two assets, we will explore simulation-based validation to test multi-asset coordination in larger deployments. This includes using virtual AAS instances and simulated MQTT messaging to evaluate latency, message congestion, and synchronisation patterns across dozens or hundreds of virtual assets. These simulations will allow us to assess the performance of our architecture at scale and identify possible bottlenecks before full physical deployment. Finally, following the trend of AAS implementations expanding beyond single-factory environments [30], our objective is to build federated and decentralised AAS architectures in which multiple factories can collaborate through shared digital twin platforms while maintaining local autonomy and data privacy.

Beyond the demonstrated use cases, deploying this framework in real manufacturing environments requires consideration of additional operational factors. Although scalability is partially addressed through modular AAS design and edge communication, maintenance and lifecycle management must also be considered. Using standardised submodels, asset-specific data structures can be reused and modified without re-engineering the system architecture, enabling more efficient updates and replacements. In addition, the framework can support central monitoring tools to track asset health and communication status, helping to plan predictive maintenance. Regarding cybersecurity, the system would benefit from adding role-based access control (RBAC) and submodel-level permissions to restrict sensitive operations and enforce access boundaries [41]. These enhancements are essential for secure industrial-scale deployments where asset coordination is distributed and high-volume data flows are subject to strict security and integrity constraints.

## Conclusion

The proposed framework advances Industry 4.0 ecosystems by enabling bidirectional, real-time interaction between assets and their digital counterparts through structured AASs. The framework addresses the interoperability challenges inherent in traditional DT implementations by integrating REST APIs for deterministic data retrieval and MQTT for event-driven communication. Experimental validation across two industrial use cases, synchronised velocity control of KUKA robots, and event-driven task coordination between a KUKA TITAN and a MiR 250, demonstrated the framework’s capability to reduce reliance on centralised control systems. The seamless integration of heterogeneous assets (e.g., industrial robots and AGVs) underscores its practicality in dynamic manufacturing environments.

The modular AAS architecture combined standardised headers for cross-platform identification with customisable submodels tailored to asset-specific functionalities (e.g., navigation data for AGVs and programme states for robotic arms). This design enabled legacy systems such as the KUKA KR1000 to interoperate with modern AGVs via a PostgreSQL-based registry, mitigating the transitional barriers observed in monolithic architectures. Furthermore, the dual-protocol approach addresses communication silos, ensuring that both structured queries (via REST) and real-time exchanges (via MQTT) coexist within a unified framework.

However, scalability tests can result in latency spikes during concurrent MQTT message bursts, highlighting the need for edge computing to decentralise data processing in large-scale deployments [34]. Although basic TLS encryption and API authentication were implemented, expanding cybersecurity to include submodel-level access control remains critical, given vulnerabilities in bidirectional IIoT protocols [41]. Additionally, reliance on custom middleware for proprietary systems (i.e., KUKA’s KRL) emphasises the need for vendor-neutral semantic templates to streamline adoption.

Future work will focus on three areas: (1) deploying edge-native AAS instances to minimise cloud dependency, (2) developing ontology-driven submodel templates for standard asset classes (i.e., CNC machines, PLCs), and (3) embedding machine learning directly within submodels for predictive parameter adjustments. These enhancements transition the framework to proactive synchronisation and co-optimisation, where assets autonomously negotiate workflows based on shared submodel insights.

This work lays a foundation for Industry 4.0’s vision of smart manufacturing by bridging the gap between static DTs and adaptive cyber-physical systems.

## Figures and Tables

**Fig. 13:**
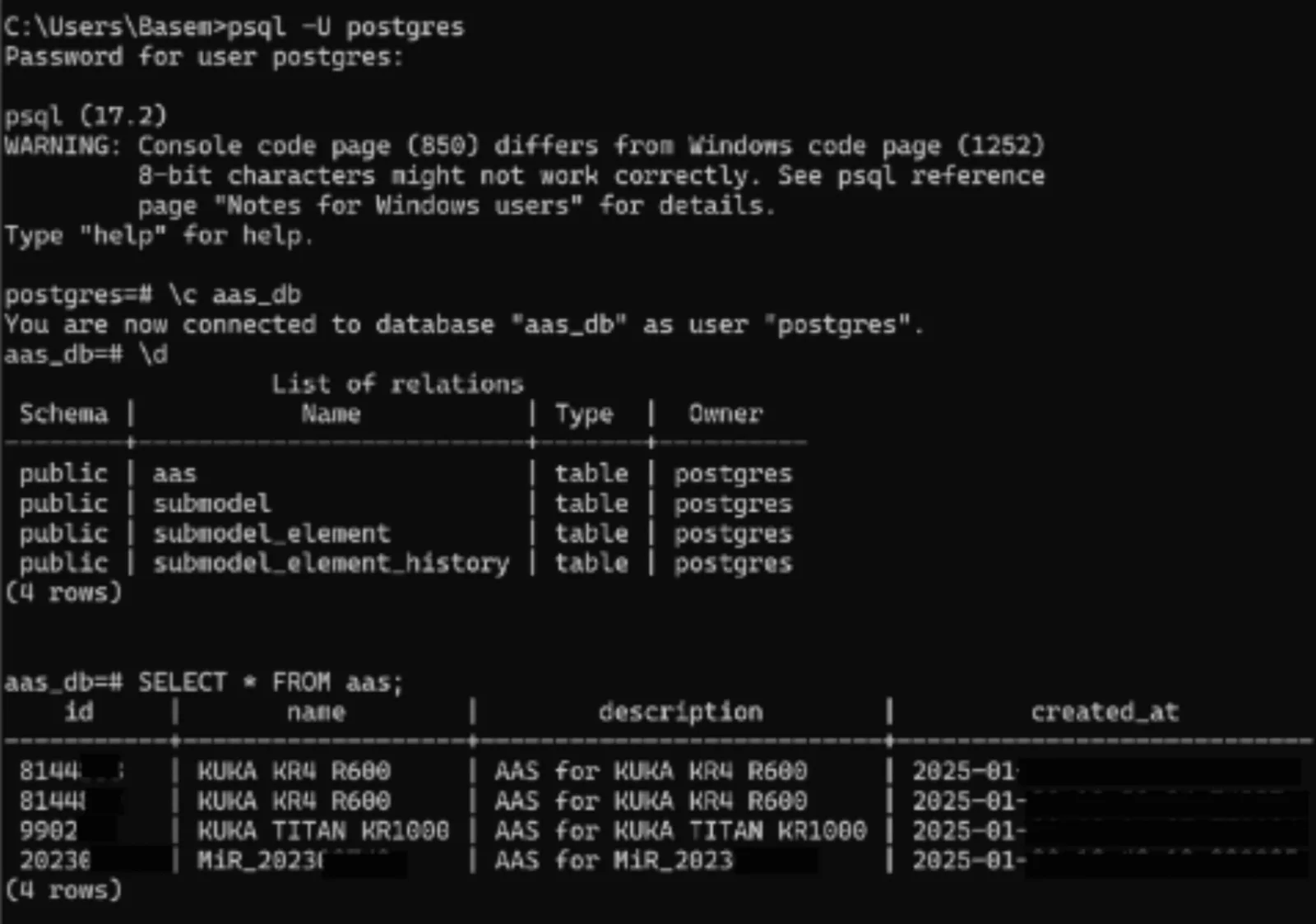
Database in PostgreSQL with created tables to host AAS data

**Fig. 14:**
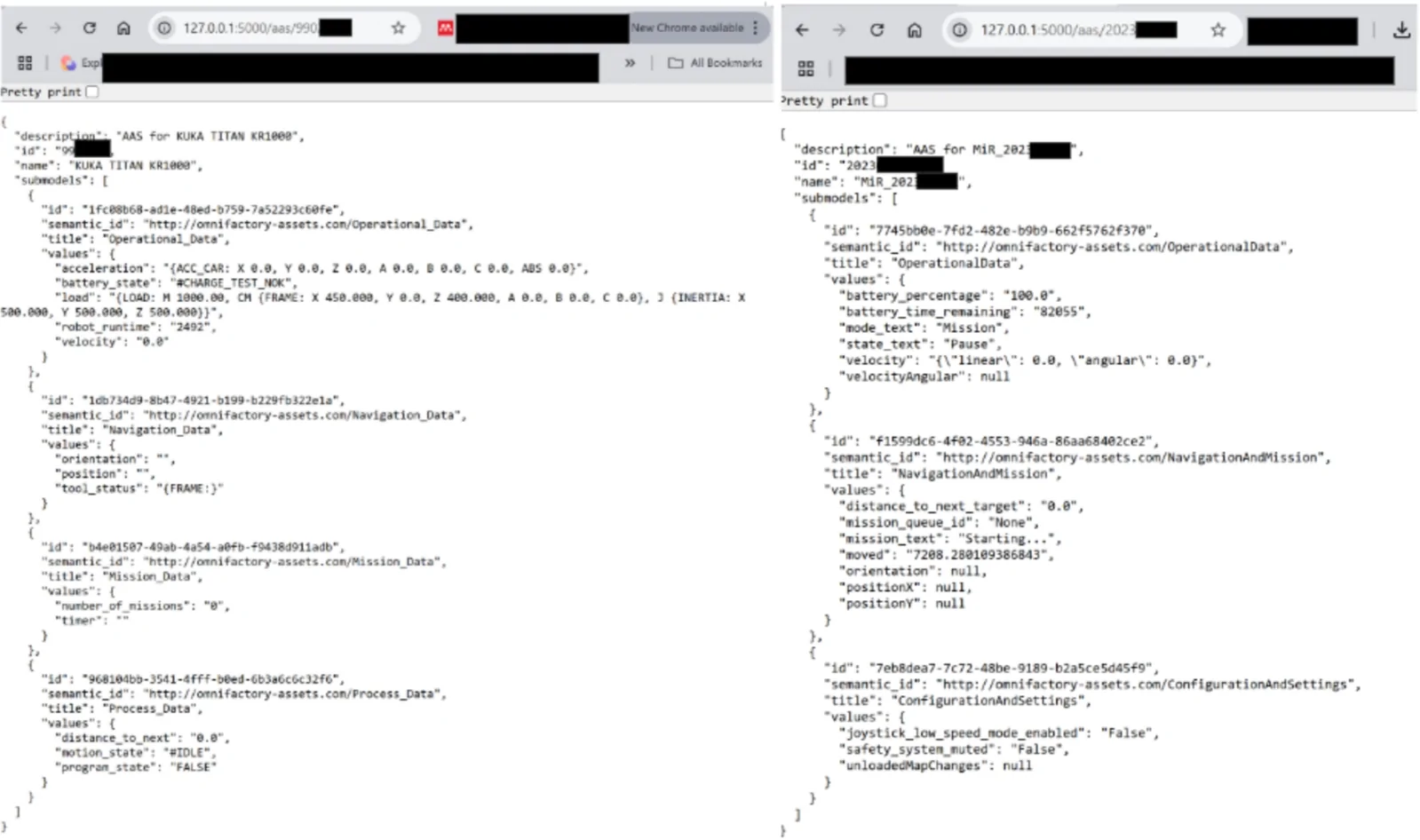
Live AAS of KUKA Titan robot (left) and MiR (right) queried from the AAS server

**Fig. 15:**
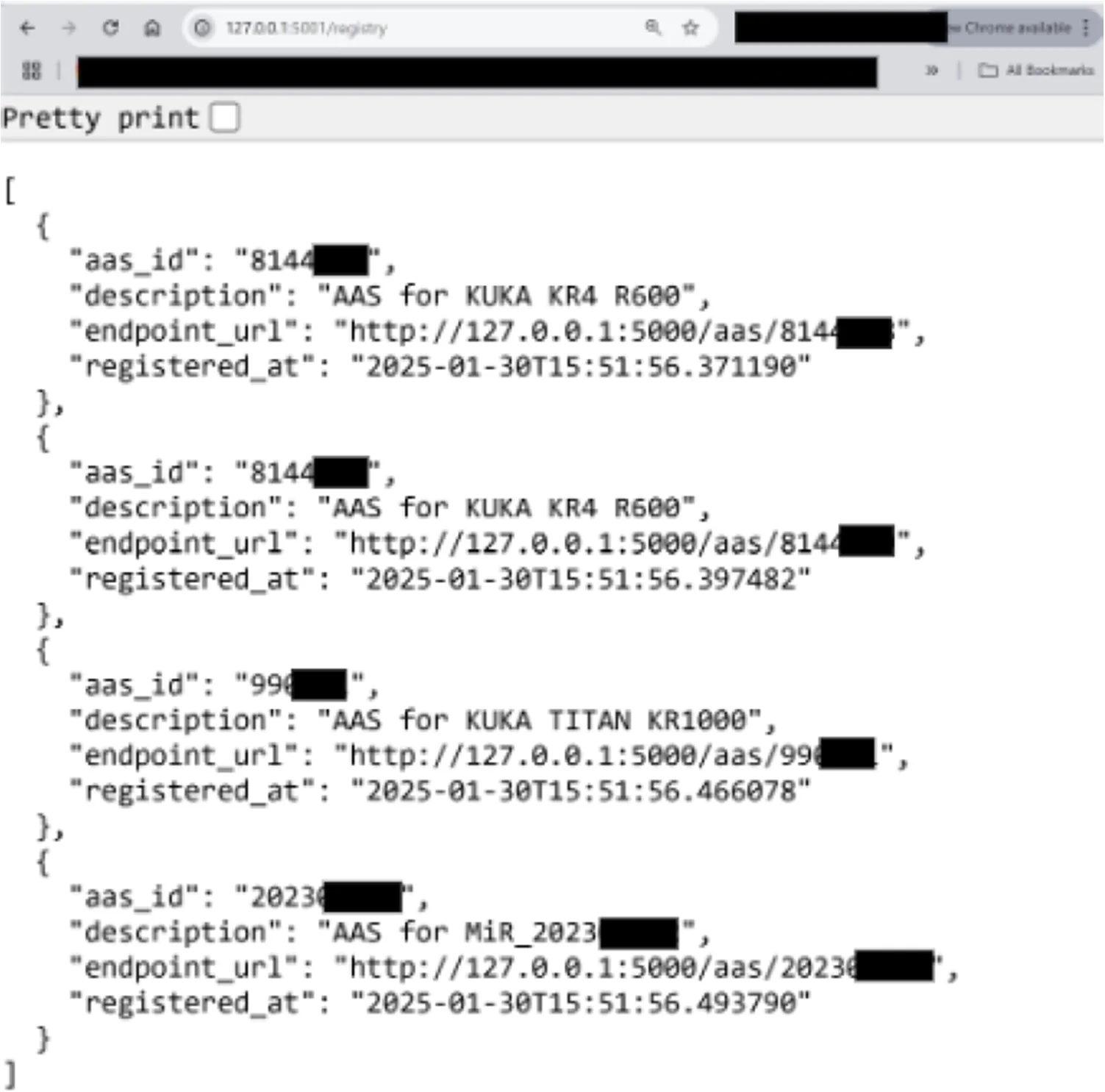
List of registered AASs and their details from the AAS Registry

## Appendix A: Section title of first appendix

An appendix contains supplementary information that is not an essential part of the text itself but may help provide a more comprehensive understanding of the research problem, or that is too cumbersome to include in the body of the paper (Figs. 13, 14 and 15).
